# Prevalence of Excess Sodium Intake and Their Corresponding Food Sources in Adults from the 2017–2018 Brazilian National Dietary Survey

**DOI:** 10.3390/nu14194018

**Published:** 2022-09-28

**Authors:** Paula Victória Félix, Michelle Alessandra De Castro, Carlos Alberto Nogueira-de-Almeida, Mauro Fisberg

**Affiliations:** 1 Department of Nutrition, School of Public Health, University of São Paulo, Sao Paulo 01246-904, Brazil; 2 School Feeding Coordination, São Paulo City Hall, Sao Paulo 01069-900, Brazil; 3 Medical Department, Federal University of São Carlos, Sao Carlos 13565-905, Brazil; 4 Instituto Pensi, Fundação José Luiz Egydio Setubal, Hospital Infantil Sabará, Sao Paulo 01227-200, Brazil

**Keywords:** sodium, salt, nutritional epidemiology, dietary survey, nutrient intake

## Abstract

Excessive sodium intake has been related to high blood pressure, a central risk factor for cardiovascular disease. In the present work, updated estimates of sodium intake, the main food sources, and the prevalence of excessive intakes in a nationwide multi-ethnic sample of Brazilian adults (2017–2018 Brazilian National Dietary Survey) were presented. Based on two 24-h recalls adjusted for the within-person variation, the overall median of sodium intake was 2432 mg. The prevalence of adults exceeding the recommended limits (tolerable upper limit, UL, and the chronic disease risk reduction cut-off point, CDRR) was 61% and 56%, respectively. The median sodium intake and proportion of individuals above the limits varied according to the subgroups evaluated. Males and adults (20–29 years) presented the highest excessive sodium intakes, and consequently, lowest adherence rates to UL and CDRR recommendations. The top food sources of sodium were white bread and toast (12.3%), beans (11.6%), white rice (10.6%), beef (7.7%), and poultry meat (5.5%). Given the health benefits of dietary sodium reduction, it would be advisable to enhance the current national initiatives of awareness and educational campaigns’ combined efforts to reduce sodium in processed foods to effectively decrease this micronutrient intake across the Brazilian population.

## 1. Introduction

Numerous studies provided ample evidence that excessive dietary sodium intake is the top risk factor for cardiovascular diseases (CVD), such as heart failure, stroke, and hypertension (HTN), which are the major causes of death and disability in the world [1,2]. Globally, a quarter of all diet-related deaths and disability-adjusted life years (DALYs) were attributable to a high intake of sodium (being responsible for 2 million [95%CI 0.5–4] deaths and 45 [95%CI 1395] million DALYs in 2019) [3]. Reducing salt intake in populations is considered the most cost-effective intervention or even cost-saving approach to decrease the burden of non-communicable diseases (NCD), endorsed by the World Health Organization (WHO), as the primary strategy to prevent CVD [2,4]. 

Based on these findings, the National Academies of Sciences, Engineering, and Medicine (NASEM) established the Chronic Disease Risk Reduction (CDRR) intake, a chronic-disease-specific recommendation for dietary sodium of 2300 mg/day [5]. Although the 2019 sodium CDRR intake is equivalent in number to the tolerable upper limit (UL) released in 2005, the UL was intended to provide guidance on safe intake levels, not to serve as an intake goal [6]. Limiting sodium intake to recommended parameters is expected to reduce chronic disease risk among healthy persons, primarily by lowering blood pressure [5]. These recommendations are a set of quantitative reference values developed jointly for the United States and Canada, and since many countries do not have national recommendations, they are the most widely used values globally for recommended intakes of essential nutrients [6]. 

Brazil has committed to achieving the global target of reducing population salt intake by 30% by 2025 [7]. In 2011, the Ministry of Health launched the 2011–2022 Strategic Action Plan for Tackling NCD, which aims to cope with and restrain NCD, including hypertension, diabetes, cancer, and chronic renal disease. One of the targets was to reduce the average salt consumption by promoting intersectoral actions, such as voluntary agreements with the food industry, health promotion in school and work settings, healthy food regulations, and healthcare services [8]. Despite long-standing recommendations to limit sodium [9] and efforts from government programs [8], the current health policies have not effectively reduced dietary sodium in the Brazilian population. A previous publication from the Brazilian National Dietary Survey shows that the prevalence of excess dietary sodium intake has not changed over the past decade, ranging from 54.4% in 2008 to 53.5% in 2018 [10]. A decrease was observed in the proportion of food products that had a sodium content within the established goals [11]. In addition, accurate measurement of sodium intake presents enormous challenges due to the extensive distribution of sodium in foods, preparation methods, and the widespread use of sodium compounds in food processing. Since the market changes continuously, food sources of sodium can also change, highlighting the need to monitor data on salt consumption at the population level and provide essential information to policymakers and all interested stakeholders on the implementation, progress, limits, and effects of a sodium reduction policy [12]. 

Nevertheless, particularly in an ethnically diverse country, such as Brazil, a better understanding of dietary patterns and actual sodium intake is crucial to patient counseling and program planning. Thus, this study aimed to (i) describe the prevalence of excess sodium intake in the context of the CDRR and UL intake goals and (ii) provide information on the current sources of dietary sodium in a representative Brazilian population using intake data from the 2017–2018 BNDS.

## 2. Materials and Methods

### 2.1. Study Design and Population

The general data were obtained from the 2017–2018 Brazilian National Dietary Survey (BNDS) and the Household Budget Survey (HBS), the latest available edition. The HBS is a survey carried out by the Brazilian Institute of Geography and Statistics (IBGE, Instituto Brasileiro de Geografia e Estatística), the official agency of Brazilian Populational Statistics, and designed to collect data on consumption expenditure, life conditions, as well as nutritional information in a representative sample of Brazilians. In summary, the 2017–2018 HBS used two-stage cluster sampling; in the first stage, the census tracts (primary sample units) were randomly selected from each stratum of census tracts. In the second stage, permanent private households (second sample units) were randomly selected without replacement within census tracts. More details about the sampling plan are obtained elsewhere [10]. 

Of the 57,920 households sampled, a subsample of 20,112 households (~35% of the total sample) was randomly selected for data collection on individual food intake (see Supplementary Figure S1). Thus, the final sample included 28,153 adults (20–59 years, both sexes, non-pregnant, non-lactating) with sociodemographic, life condition, and dietary data collected [13]. The present study was conducted according to the guidelines laid down in the Declaration of Helsinki, the Brazilian Resolution Number 196/96 on research involving human subjects, and under Brazilian Law #5534 from 14 November 1968, which guarantees the confidentiality of the information collected by all national census and surveys.

### 2.2. Sociodemographic and Anthropometric Information 

Individuals’ information on age, sex, geographic region (North, Northeast, Southeast, South, Midwest), household area (urban/rural), self-reported ethnicity, dietary habits, per capita family income, and educational level (years of schooling) was collected by a structured questionnaire administered by trained interviewers in the household. 

Self-reported ethnicity was based on Brazilian law #12711 from 29 August 2012, which provides for admission into public universities and institutions and was categorized as White, Asian, Black, Mixed-race, or Native. Per capita family income was estimated by summing all monetary and non-monetary income reported by family members divided by the number of family members, and minimum wage was 954.00 Brazilian Real (BRL) in 2018 (equivalent to USD 298.53, 1 USD = 3.1957 BRL on 15 January 2018). 

Body mass index (BMI) was estimated based on self-reported weight and height information and was classified as without excessive body weight (BMI < 25 kg/m^2^), and with excessive body weight (BMI ≥ 25 kg/m^2^) [14]. 

Food security status was measured by the Brazilian Food Insecurity Scale (EBIA, Escala Brasileira de Insegurança Alimentar), an adapted scale from that proposed by the United States Department of Agriculture (USDA) and validated to the Brazilian population [15,16]. The higher number of affirmative responses indicates greater food insecurity. The final score is categorized as food security (score 0 or no affirmative responses), mild food insecurity (1–5 points for households with members aged < 18 years, and 1–3 points for households without members aged < 18 years), moderate food insecurity (6–9 and 4–5 points for households with members aged < 18 years and households without members aged < 18 years, respectively), and severe food insecurity (10–14 and 6–8 points, respectively) [15].

### 2.3. Dietary Data 

Dietary data were collected by two non-consecutive 24 h dietary recalls (24HR) throughout all days of the week and seasons of the year. Both 24HR were collected by face-to-face interviews at participants’ homes following procedures described in the USDA Automated Multiple Pass Method [17]. In addition to 24HR, participants were asked if they consumed or followed a specific diet for health purposes (e.g., for obesity, diabetes, high blood pressure, hypercholesterolemia, or other health condition). 

Individuals were instructed by interviewers to inform them of all consumed foods and beverages in household measures and report the mealtime, place of consumption (i.e., at home or away from home), cooking methods, and added seasonings. The dietary data were entered into the software developed by IBGE that automatically converted the household measures into standard weight and volume measures, such as grams and milliliters. Quality control of the 24HR was conducted during and after the interview to identify and correct misreporting in real time. 

The energy and nutrient content of each food item reported in 24HR was obtained by the Brazilian Food Composition Table (TBCA-USP), version 7.0, developed by the Food Research Center (FoRC) at the University of São Paulo (USP), available at http://www.fcf.usp.br/tbca (accessed on 15 July 2022), in accordance with standards and guidelines for generation, compilation, and use of food composition data of FAO/INFOODS (food and agriculture organization/international network of food data system). 

Sodium intake was adjusted for within-person variation through the web-based statistical modeling technique multiple source method (MSM), version 1.0.1, updated in 2020. The MSM was developed within the European food consumption and validation project as a suitable technique for estimating the usual nutrient and food intakes (including those episodically consumed) based on two or more short-term dietary methods per individual, such as the 24HR [18,19]. The effects of day-of-the-week (weekday vs. weekend) and atypical day of dietary intake (no vs. yes) were considered as adjustments in the models. 

### 2.4. Food Grouping 

The 1508 different foods reported in both 24HR were classified into 54 mutually exclusive food groups. Foods were combined based on the frequency of consumption, similarity of the nutrient profile, dietary habits, and culinary usage in the Brazilian population. The total sodium intake comprised the sodium naturally present in food and added during preparation (e.g., rice cooked with salt, sautéed vegetables, etc.). A detailed description of foods included in each food grouping is described in Supplementary Table S1. 

### 2.5. Statistical Analyses 

This report was prepared following the strengthening the reporting of observational studies in epidemiology-nutritional epidemiology (STROBE-Nut) statement specified for nutritional epidemiologic studies [20]. Descriptive analyses of median, percentage, and 95% confidence intervals (95% CI) were performed using Stata^®^ software (version 14.0, 2011, Stata Corp LP, College Station, TX, USA) considering the complex sampling design and significance level of 5%. Differences in socioeconomic, demographic, anthropometric, and lifestyle variables were tested by Theil–Sen median test for complex sampling design. The post hoc Dunn test was used to identify the significance between groups. The population prevalence exceeding dietary sodium intake was estimated according to the NASEM parameters UL (UL = 2300 mg/day) and CDRR (reduce intakes if above 2300 mg/day) for adults ≥ 19 years old [5]. 

The organizational contribution of each food group to the sodium intake was determined using the method proposed by Block et al. [21]. This method estimates the corresponding percentage of foods or food groups consumed by the population from the total nutrient intake. Individuals who reported intake of a certain food group in at least one 24HR were classified as “consumers” regardless of the amount reported. The prevalence of consumers and sodium density were calculated for food groups contributing to >1% of total sodium intake.

## 3. Results

Sociodemographic, lifestyle, and dietary characteristics of all included participants, their sodium consumption, and the prevalence of excess sodium intake are shown in Table 1.

### 3.1. Study Population Characteristics

The sample is predominantly composed of adults living in urban areas (86.3%), in the Southeast region (42.8%), having per capita family > 1 minimum wage (40.4%), with schooling above high school (60.2%). Most of the population reported being Black, mixed race, or native (56.7%) and presented excessive body weight (55.6%). Regarding dietary characteristics, most participants reported not being on a diet (86.9%) and not having the habit of adding extra salt to already prepared dishes (85.5%). The EBIA classification indicates a significant portion of the population in some degree of food insecurity (40.5%). Similar proportions were found for variables sex and age group.

### 3.2. Dietary Sodium Intake 

Overall median sodium consumption of Brazilian adults was 2432 mg (95%CI: 1902–3074). As to the regions of Brazil, we found lower intake in the North region (2223 mg; 95%CI: 1702–2880) and higher in the South (2485 mg; 95%CI: 1941–3166). Sodium consumption declined with advancing age. The median intake was 2595 mg (95%CI: 2029–3261) for individuals aged 20–29 years and 2449 mg (95%CI: 1912–3126) for individuals aged 30–39 years. For individuals aged 40–49 years and 50–59 years, sodium intake was 2382 mg (95%CI: 1889–3025) and 2306 mg (95%CI: 1799–2912), respectively (*p* < 0.01 for all comparisons). Median sodium consumption was greater in males (2769 mg; 95%CI: 2176–3431; the highest consumption found), also among those with per capita family income above one minimum wage (2471 mg; 95%CI: 1927–3115), that had an educational level above high school (2461 mg; 95%CI: 1934–3105), that reported not following a specific diet (2491 mg; 95%CI: 1954–3129), declared the habit of adding extra salt to already prepared meals (2529 mg; 95%CI: 1979–3195), and individuals classified as in food security status (2466 mg; 95%CI: 1939–3109). Household area, self-reported ethnicity, and BMI status did not significantly differ in sodium intake.

### 3.3. Prevalence of Excessive Sodium Consumption 

The percentage of Brazilian adults whose sodium intake exceeded the recommended limit considering the UL and CDRR cut-off points was 61.0% and 56.1%, respectively. The lowest prevalence of excessive sodium consumption was found in individuals who reported following a specific diet (UL: 45.6%; CDRR: 38.9%) and females (UL: 48.0%; CDRR: 42.2%), whereas the highest prevalence of excessive consumption was found among males (UL: 71.6%; CDRR: 70.1%) and individuals aged 20–29 years (UL: 65.9%, CDRR: 62.5%). 

### 3.4. Main Food Sources of Sodium

The 25 food groups that collectively accounted for over 90% of the dietary total sodium intake and their prevalence of consumers are presented in Table 2. White bread and toast were the main sources of sodium intake (12.3%), emphasizing French bread, which contributed to 9.3% of sodium intake—followed by beans (11.6%), white rice (10.6%), beef (7.7%) and poultry meat (5.5%). These top five food groups accounted for 47.6% of total sodium intake, and they are also foods widely consumed by the population, with more than 50% of individuals reporting their consumption. Conversely, the traditional food items that presented high sodium density, such as cured meats (1781 mg/100 g) and sausages (1460 mg/100 g), did not emerge as major sodium contributors.

In the overall population, the top 10 food groups with the highest proportion of individuals reporting consumption in at least one recalled dietary data were water (86%), coffee (85%), white rice (83%), sugar and honey (78%), beans (72%), white bread and toast (61%), leafy and non-leafy vegetables (57%), beef (56%), juices (50%), and poultry meat (48%). The complete table with all food groups and their prevalence of consumers can be found in Supplementary Table S1.

## 4. Discussion

This study revealed that most Brazilian adults consume dietary sodium above UL and CDRR recommendations and could benefit from lowering sodium intake to reduce their cardiovascular disease risk. The main food sources of dietary sodium were traditional foods widely consumed by the population, such as white bread and toast, beans, white rice, beef, and poultry meat. 

Epidemiologic studies indicated a rapidly growing prevalence of HTN in Brazil. According to the 2013 National Health Survey (PNS), conducted in partnership with the Ministry of Health and IBGE, the prevalence of self-reported HTN in Brazil was 21.4%; more recently, the PNS 2019 recorded this proportion at 23.9% [22]. Simulation studies carried out in Latin America have estimated that reducing sodium consumption as recommended by the WHO could reduce about 47,000 deaths from cardiovascular diseases, the equivalent of 85 million dollars in health care [23]. With the increasing prevalence of HTN, these updated data on dietary sodium contributors that may influence high blood pressure are essential to developing individualized and population-wide strategies. 

Overall, dietary sodium intake was notably higher than the NASEM parameters UL and CDRR. In the overall sample and among the most subgroups evaluated, sodium intake remained persistently above recommendations, surpassing the prevalence observed in HBS’s previous editions [10]. We found substantial differences in the proportion of adults meeting sodium intake recommendations. In general, UL parameters resulted in higher percentages of individuals exceeding sodium intake than CDRR. Increased cardiovascular disease risk due to excess intake was found mainly in men and individuals in the age category 20–29 years old, which is consistent with previous Brazilian studies that described these subgroups as the greatest consumers of salt [24,25] and having worse diet quality [26,27]. On the contrary, women and older individuals are recognized as having more health concerns, seeking out medical assistance regularly, and following prescribed treatment more accurately than men and young people [28,29], which can partially explain the lower estimates expressed by this subgroup. 

Differences in the prevalence of excess sodium intake among Brazilian macro-regions may reflect the well-known country’s social and economic inequalities that are associated with food security condition. The FAO defines food insecurity as “when people lack secure access to sufficient amounts of safe and nutritious food for normal growth and development and an active and healthy life” [30]. In our study, individuals facing moderate or severe food insecurity presented lower sodium consumption. According to the literature, this scenario can also occur for several other micronutrients [31,32]. In agreement, the lowest sodium consumption was found in the North region, which has the higher proportion of the population facing moderate and severe food insecurity (29.3%), whereas the South region showed a higher percentage of individuals exceeding sodium intake and fewer households in moderate or severe food insecurity (4.9%) (Supplementary Table S2). 

The detailed dietary data in the current study allowed us to identify the key dietary sources of sodium in Brazilian adults. Food groups that most contributed to total sodium intake were white bread and toast, followed by beans and white rice. These are typical Brazilian foods that are not generally considered primary dietary sodium sources. However, the frequency of their consumption renders them important contributors to total sodium intake for the study population. For example, 100 g of beans presented 302 mg of sodium, whereas 100 g of boiled white rice had 289 mg of sodium. These amounts are considered low compared to other food groups consumed by the population, such as sausages and hot dogs (1460 mg per 100 g) and pizza and calzones (847 mg per 100 g). For the overall population evaluated, these high-sodium foods are eaten by fewer people than staple foods, such as white rice and beans. However, it should be considered that the consumption of high-sodium foods can reach 50% of the recommended intake just from a single portion. 

The present study indicates discretionary salt as the leading source of sodium following previous data from 2008–2009 BNHS, which estimates that nearly three-quarters (74.4%) of total sodium intake came from table salt and salt-based condiments in the Brazilian population [33]. These findings are in agreement with a systematic review that included 80 studies from 34 different countries [34], whose main results demonstrate that in developing countries, the predominant source of sodium is salt added as part of the traditional recipes, and the food components “bread and bakery products”, “cereal and grain products”, “meat”, and “dairy products” appeared to be the main global contributors to sodium daily intake. 

Recognizing that bread products are relevant sources of dietary sodium in the Western diet, some countries in Latin America have set voluntary or regulated targets and timelines for reducing sodium content in bread, among other food products. In Argentina, where this food group accounts for almost 25% of total salt in the diet [35], over 90% of the farinaceous products had sodium content below the mandatory targets [36]. Costa Rica has reported gradual changes in meeting the national targets, with 69% of bread meeting the recommendations [37]. Moreover, a study performed in 14 countries of Latin America and the Caribbean met an 82% general compliance with the regional targets [38]. White bread and toast are two foods commonly consumed at breakfast and intermediary meals, such as snacks, in Brazil. In the previous study with the same sample from the 2017–2018 BNDS, Castro et al. (2022) identified that the dietary pattern “Brazilian breakfast style” was characterized by white breads and toasts, butter and margarine, table sugar, coffee, and natural juice [39]. It is possible that the high frequency of consumption of white bread and toast (61% of the population reported the consumption of this food group) and their high sodium content (i.e., ranging from 430 mg/100 g of homemade bread to 712 mg/100 g of toast), may explain this food group on the top of the dietary sources of sodium intake among Brazilians.

Reducing the population’s sodium intake has been on the health agenda in Brazil for many years and requires a combination of strategies to address all dietary sources of sodium [40]. For instance, the proportion of dietary sodium obtained from salt added by individuals during cooking is relevant and interventions aiming to nutrition education (i.e., salt substitution) may be effective in reducing the sodium content of many food preparations, such as rice, beans, and meat. Moreover, reformulation strategies may be effective in settings where a large proportion of dietary salt comes from packaged foods and foods prepared outside home [34]. According to the 2018 Brazilian Ministry of Health official monitoring report, the food categories “loaf bread” and “commercial buns” showed more than 95% adequacy of sodium content. When considering all food categories included in the voluntary agreement, the reported adequacy was 87% [11]. 

Since sodium intake in Brazil remains high despite the positive progress described, findings from the present study reinforce the need to also support campaigns focused on homemade preparations and bakery foods (which are broadly consumed by the population and the major contributors of sodium) to have a greater impact on sodium intake and improve CVD outcomes more effectively. Some common policies already in progress include intersectoral efforts in the education and communication fields, such as the elaboration of the dietary guidelines for the Brazilian population, which presents qualitative orientations regarding moderate sodium intake in strategic environments [41]. Even though packaged food products are not the main source of sodium in the population’s diet, reducing sodium in processed foods is also needed [40]. To address the central problem of nutrition facts labeling, which is the difficulty of interpreting and understanding nutrition information by the population, the Brazilian Health Regulatory Agency (ANVISA) issued RDC Resolution n. 429/2020 and Normative Instruction n. 75/2020 on nutrition labelling requirements [42,43]. The purpose is to make more visible the disclosure of ingredients that may represent a health risk, establishing front-of-pack labelling requirements for food and beverages with high quantities of sodium, fat, or sugars. For sodium content, the application of the front-of-pack labelling is expected when sodium ≥ 600 mg per 100 g, or ≥300 mg per 100 mL. The resolution come into force on 9 October 2022 and will help consumers to make informed choices.

Given the current status of salt intake in the country, other strategies must be reinforced to promote healthy diets and environments. It becomes essential to support massive campaigns that encourage the reduction in sodium consumption, especially regarding the addition of table salt and condiments during cooking, concerning specific cultural cooking practices. In contrast, the addition of herbs and spices can highlight the salty taste and improve the flavor and nutritional characteristics of foods, favoring the acceptance of these foods with reduced salt content. Additionally, it is necessary to propose policies more appropriate to regional realities, which favor the optimization of investments in Brazil’s Unified Health System and decrease inequalities among states. 

Our study included the most recent available data on a large nationally representative sample of Brazilians and provided up-to-date evidence on sodium intake and its food sources. In addition, to our knowledge, no prior studies have analyzed adherence rates to evolving CDRR recommendations for sodium consumption in the Brazilian sample. 

However, the present study has some limitations. The ranking of food categories by the contribution to sodium consumption depended on methods used to categorize specific foods and consumption frequency, which may lead to different results depending on the compositional criteria of each food group. In addition, were used the UL and CDRR parameters estimated for the North American and Canadian populations since there are no national recommendations aiming to guide safe sodium intake level and/or reduce the risk of NCD. Additionally, we estimate intake with 24HR; this information does not come from a direct biomarker, such as 24-h sodium urine excretion (reference method). Compared to the previously published estimate of mean salt intake measured from 24-h urine samples using a Brazilian population sample of 8083 adults from the 2013 HBS [24], the mean salt intake estimated using 24HR was lower. The 24HR tends to underestimate the intake of nutrients due to the lack of precision and memory bias of the participant since this method is complex, and participants tend to change the consumption report in the dietary recall interview. Nevertheless, it is a useful tool for identifying sources of sodium in diets and a reasonable approach to estimating sodium intake in large epidemiologic studies [44]. 

The population information reported in this study is essential to approaching sodium consumption in Brazil. As this country experiences the effects of a nutrition transition toward pre-prepared and processed foods, ongoing monitoring of dietary sources, estimated salt intake, and data on the knowledge and behaviors on salt intake will be essential to monitor changes and refocus interventions throughout the lifespan of a national action plan for salt reduction.

## 5. Conclusions

National programs and interventions to reduce sodium intake and promote healthy food options remain essential. Sodium intake in Brazil exceeded the recommended limit considering the UL and CDRR cut-off points. Higher medians and prevalence of excessive sodium consumption were found among males and individuals aged 20–39 years old. The food groups that most contributed to total sodium intake were traditional foods widely consumed by the population, such as white bread and toast, beans, white rice, beef, and poultry meat. 

Urgent action is needed to implement a program to achieve the WHO salt reduction target of 30% by 2025. Therefore, improving the quality and nutritional composition of foods, as well as moderating consumption of certain food groups, are crucial goals for health authorities and achieving a balanced diet in the Brazilian population. The data presented can be used to improve public health policies aimed at reducing the sodium content of the diet, which may help reduce the prevalence of hypertension-related diseases.

## Figures and Tables

**Table 1:** Median sodium intake and prevalence of excessive sodium consumption according to socioeconomic, demographic, and anthropometric characteristics of the Brazilian adult population. Household Brazilian Budget Survey, 2017–2018 ^1^.

Characteristics	Total Population		Sodium Intake (mg)		*p*-Value ^3^	% Exceeding Sodium Intake According to	
	n	% (95%CI)	Median	IQR		UL	CDRR
Overall population	28,153	100	2432.4	(1902.7, 3073.8)		61.0	56.1
Geographic region							
North	4132	8.2 (7.7, 8.7)	2222.9 ^abcd^	(1701.6, 2880.0)		52.8	45.9
Northeast	9717	26.4 (25.6, 27.3)	2462.2 ^c^	(1901.1, 3151.9)		62.5	57.4
Southeast	7029	42.8 (41.7, 44.0)	2431.1 ^bc^	(1927.4, 3026.3)		60.6	56.5
South	3699	14.6 (13.9, 15.4)	2485.5 ^ab^	(1940.6, 3166.2)		62.9	58.2
Midwest	3576	7.9 (7.5, 8.4)	2438.2 ^d^	(1882.4, 3082.6)	0.014	60.2	55.6
Area							
Urban	21,863	86.3 (85.7, 86.9)	2439.8	(1909.1, 3074.8)		61.0	56.4
Rural	6290	13.7 (13.1, 14.3)	2389.9	(1863.3, 3064.4)	0.062	59.9	54.6
Age group, years							
20–29	6665	25.1 (24.3, 26.0)	2595.5 ^a^	(2029.0, 3260.9)		65.9	62.5
30–39	7598	26.9 (26.0, 27.9)	2448.8 ^a^	(1911.9, 3125.6)		61.8	57.3
40–49	7274	25.1 (24.2, 26.0)	2382.5 ^a^	(1889.3, 3024.8)		59.1	53.6
50–59	6616	22.8 (22.0, 23.7)	2306.2 ^a^	(1799.5, 2912.4)	<0.001	55.6	50.6
Sex							
Male	13,338	49.8 (49.2, 50.4)	2769.0	(2176.5, 3431.4)		71.6	70.1
Female	14,815	50.2 (49.6, 50.8)	2171.8	(1717.8, 2668.8)	<0.001	48.0	42.2
Self-reported ethnicity							
Mixed-race	14,532	45.1 (44.0, 46.2)	2553.2	(1897.6, 3053.7)		60.2	55.7
White	10,351	42.6 (41.4, 43.8)	2591.1	(1924.5, 3089.6)		61.7	56.5
Black	2963	11.2 (10.5, 11.9)	2564.9	(1883.7, 3089.8)		61.0	56.7
Asian	145	0.6 (0.4, 0.9)	2420.6	(1951.3, 3058.1)		56.0	44.9
Native	141	0.4 (0.3, 0.1)	2384.9	(1776.4, 2996.6)	0.060	54.0	51.6
Per capita family income ^2^							
≤1 minimum wage	13,218	40.4 (39.1, 41.7)	2374.4	(1872.5, 3016.0)		59.1	54.1
>1 minimum wage	14,935	59.6 (58.3, 60.8)	2471.8	(1926.6, 3115.1)	<0.001	62.2	57.5
Education level							
≤9 years of schooling(below elementary school)	12,702	39.8 (38.7, 40.9)	2384.3	(1847.9, 3029.6)		59.1	53.8
>9 years of schooling(above high school)	15,451	60.2 (59.1, 61.3)	2461.5	(1934.4, 3105.7)	<0.001	62.2	57.6
Body Mass Index							
Without excessive body weight	12,676	44.4 (43.4, 45.4)	2413.8	(1908.6, 3061.8)		60.6	55.4
With excessive body weight	15,477	55.6 (54.6, 56.5)	2449.5	(1898.7, 3087.0)	0.479	61.0	56.7
Followed a specific diet							
Yes	3778	13.1 (12.4, 13.8)	2081.3	(1602.0, 2644.5)		45.6	38.9
No	24,375	86.9 (86.2, 87.6)	2491.4	(1954.1, 3129.3)	<0.001	62.9	58.7
Add extra salt at the table							
Yes	3777	14.5 (13.7, 15.4)	2528.7	(1979.4, 3194.6)		63.6	60.7
No	24,376	85.5 (84.6, 86.3)	2416.5	(1888.5, 3055.7)	<0.001	60.2	55.4
Food security status							
Food security	15,878	59.5 (58.1, 60.9)	2466.1 ^a^	(1939.2, 3109.0)		62.5	57.7
Mild food insecurity	7836	27.1 (25.9, 28.3)	2414.2 ^a^	(1892.0, 3036.4)		59.9	55.5
Moderate or severe food insecurity	4439	13.4 (12.6, 14.2)	2304.0 ^a^	(1772.6, 2981.1)	<0.001	55.9	50.2

Abbreviations: UL, upper limit intake; CDRR, chronic disease risk reduction. ^1^ All the analyses considered the sampling survey design. ^2^ One minimum wage was approximately USD 298 in 2018. ^3^ Median and interquartile range (IQR) are described, and differences were evaluated using Theil–Sen test. Post hoc Dunn’s test was applied for comparing variables with three or more groups. Medians in the same variable with the same superscript letters (a–d) are significantly different (*p* < 0.01).

**Table 2:** Description of the food groups contributing to >1% of total sodium intake, prevalence of consumers, and food group sodium density among Brazilian adults. Household Brazilian Budget Survey, 2017–2018.

Rank	Food Groups	% Total Sodium Intake ^1^	% Cumulative	% of Consumers	Food Group Sodium Density (mg/100 g)
1	White bread and toast	12.3	12.3	61.0	578
	French bread	9.51		43.7	681.9
	Loaf bread	0.29		3.8	548.5
	Homemade bread	0.14		1.5	430.8
	Other White bread (except sweet rolls)	2.17		16.0	653.8
	Toast	0.16		3.3	712
2	Beans	11.6	23.9	72.4	302
3	White rice	10.6	34.4	83.4	289
4	Beef	7.7	42.1	55.7	350
5	Poultry meat	5.5	47.6	50.2	693
6	Sandwiches	5.2	52.8	22.5	346
7	Pasta	4.7	57.5	34.7	252
8	Cookies and crackers	2.8	60.3	31.9	688
9	Rice-based mixed dishes	2.8	63.1	12.7	440
10	Sausages and hot dogs	2.7	65.8	12.3	1460
11	Cured meats	2.3	68.0	3.4	1781
12	Butter and Margarine	2.1	70.2	47.0	810
13	Corn-based mixed dishes	2.1	72.3	13.8	261
14	Salty pastries	1.9	74.2	15.4	643
15	Pork meat	1.9	76.0	11.3	250
16	Roots and tubers	1.8	77.9	22.7	54
17	Beans-based mixed dishes	1.8	79.7	7.7	298
18	Fish and Seafood	1.7	81.4	12.2	201
19	Eggs and omelets	1.7	83.1	22.5	254
20	Pizza and calzones	1.6	84.6	5.9	847
21	Leafy and non-leafy vegetables	1.5	86.2	57.2	92
22	Water	1.5	87.7	85.4	3
23	Cheese	1.5	89.1	16.6	613
24	Meat-based mixed dishes	1.3	90.4	13.3	276
25	Soups and broth	1.0	91.5	11.4	365

^1^ This analysis included 54 food categories. Food groups were ranked in descending order by contribution to total sodium intake. The list of foods included in each food grouping is described in Supplementary Table S1.

## Data Availability

Data used in the present study is made publicly available by the Brazilian Institute of Geography and Statistics, accessed on 10 July 2022 (https://www.ibge.gov.br/estatisticas/sociais/saude/24786-pesquisa-de-orcamentos-familiares-2.html?=&t=microdados). The code used in this study is available upon request.

## Supplementary Materials

The following supporting information can be downloaded at: https://www.mdpi.com/article/10.3390/nu14194018/s1, Figure S1: Sample flowchart in the 2017–2018 Household Budget Survey eligible for the present study; Table S1: Frequency of consumption and list of foods that were included in each food group for adults of the 2017–2018 Household Brazilian Budget Survey; Table S2: Geographic region distribution according to food security status based on the Household Budget Survey 2017–2018, Brazil.
