# Supplementary material for: Prevalence of Excess Sodium Intake and Their Corresponding Food Sources in Adults from the 2017–2018 Brazilian National Dietary Survey
Source: Nutrients. 2022 Sep 28;14(19):4018. doi: 10.3390/nu14194018 (PMC9571186; doi:10.3390/nu14194018)
Supplement: Supplementary file 1 [file nutrients-14-04018-s001.zip › nutrients-1932325-supplementary.pdf]

Figure S1. Sample flowchart in the 2017-2018 Household Budget Survey eligible for the present study

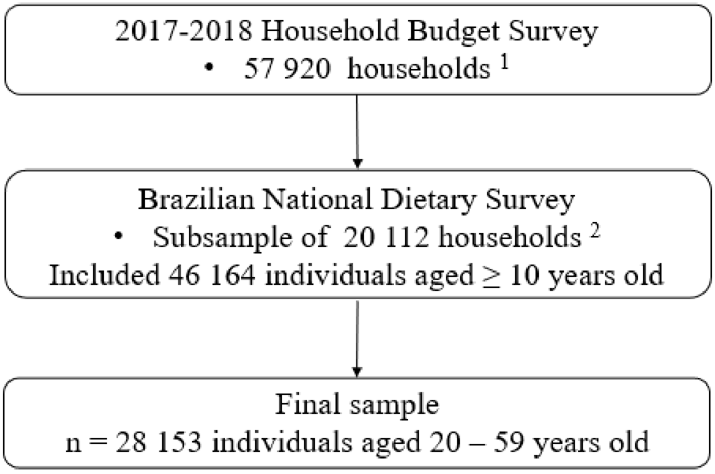

[1] Households selected at random from the pre-defined stratification system. An average loss of 15% was estimated due to possible refusals to answer the survey, and this same proportion was added to the final number of households to minimize possible losses.

[2] Households selected at random from the pre-defined stratification system.

Table S1. Frequency of consumption and list of foods that were included in each food group for adults of the 2017-2018 Household Brazilian Budget Survey.

Food Group	Food group content example	Prevalence of consumers (%)
Water	Water tap, bottled water, mineral water, sparkling water	86.2
Coffee	Brew coffee, instant coffee, cappuccino, decaffeinated coffee, espresso coffee	85.3
White rice	Cooked white rice, with or without salt	83.4
Sugar and honey	White or brown sugar, sweeteners, honey	77.9
Beans	Black beans, brown beans, white beans, pinto beans	72.4
White bread and toast	French bread, Italian bread, loaf bread, buns, toast	61.0
Leafy and non-leafy vegetables	Tomato, carrot, spinach, chayote, eggplant, okra, pumpkin, chard	57.2
Beef	Steak beef, ground beef, beef ribs (all cooking methods)	55.7
Juices	Fruit juices or commercial juices	50.2
Poultry meat	Chicken, turkey, duck, goose (all cooking methods)	48.1
Butter and Margarine	Butter and margarine (salted or unsalted, regular or light)	47.0
Fruits	Fresh fruits, fruit salad, dried fruits	36.6
Pasta	Cooked noodles, gnocchi, lasagna, cannelloni, ravioli, with or without sauce	34.7
Cookies and crackers	Cookies without fillings or toppings, salt crackers	31.9
Soft drinks	Fruit-flavored drinks, cola and noncola soft drinks	25.6
Sweets and candies	Chocolate, puddings, ice creams, sweet rolls, milk candy, peanut brittle	24.5
Roots and tubers	Potato, sweet potatoes, cassava, yam (all cooking methods)	22.7
Eggs and omelets	Chicken eggs, quail eggs, omelets (all cooking methods)	22.5
Sandwiches	Hot dog, hamburger sandwich, cold cuts sandwiches, wrap	21.1
Flours	Corn flour, tapioca flour, fried manioca flour (farofa)	20.8

Vegetables oils	Olive oil, soybean oil, sunflower oil	20.4
Cake	White cake, chocolate cake, carrot cake, sponge cake, with or without topping and filling	18.2
Cheese	White or yellow	16.6
Dairy products and beverages	Yogurt, kefir, milk smoothie, fermented dairy drinks	16.1
Salty pastries	Croquette, croissant, Italian focaccia, fried or baked pastry	15.4
Corn-based mixed dishes	Cooked cornmeal, grits, sweet corn mush, cooked hominy	13.8
Meat-based mixed dishes	Stroganoff, minced meat roulade, fish stew, stuffed chicken	13.3
Rice-based mixed dishes	Rice with chicken, risotto, fried rice	12.6
Sausages and hot dogs	Sausages, frankfurters, hot dogs	12.3
Fish and Seafood	Fish, shrimps, crab, oyster, squid, canned sardine, canned tuna	12.2
Soups and broth	Soups with or without beans, noodles, rice, corn meal and vegetables; instant soups	11.4
Pork meat	Pork chop, pork ribs, pork loin (all cooking methods)	11.3
Sauces and spices	Tomato sauce, white sauce, ketchup, mayonnaise, salad dressing, vinegar, salt, ground pepper	9.8
Tea	Green, black, herbal, chamomile tea	9.4
Other mixed dishes	Grits with ground beef, potato salad, stuffed eggplant, Shepherd's pie, cabbage rolls (with cereals, beans, legumes, meat and/or other ingredients)	9.1
Milk and milk alternatives	Whole, low-fat, skimmed, fortified milks and soy beverages	8.6
Alcoholic beverages	Fermented and distilled alcoholic beverages	7.8
Beans-based mixed dishes	Brazilian black beans stew (feijoada), cassoulet, mixed beans salad	7.7
Cold cuts	Ham, mortadella, salami, roast beef	6.7
Pizza and calzones	Calabrese pizza, mozzarella pizza, vegetarian pizza, calzones	5.9
Brown bread and toast	Rye bread, multi-grain bread and toast, whole wheat bread and toast	4.8
Organ meats	Liver, intestine, kidney, stomach (pork, beef, and poultry)	4.6
Cured meats	Bacon, jerked beef, salted meat	3.4
Popcorn	Sweet or salty, with or without butter, regular or microwaved	3.1
Brown rice	Cooked brown rice, three grain rice, seven grain rice, with or without salt	3.0

Cereals	Corn flakes, granola bars, oats, wheat, breakfast cereal, quinoa	3.0
Flavored Snacks	Potatoes chips, corn chips, flavored chips	2.6
Nuts and Seeds	Almonds, Brazil nuts, cashew nuts, pumpkin seeds, sesame seeds, linseed	2.1
Salty pies	Chicken pie, cheese pie, quiche	1.9
Other legumes	Cooked soybeans, lentils, chickpeas, snow peas	1.7
Flavored powder	Chocolate, strawberry artificially flavored powders	1.6
Other non-alcoholic beverages	Coconut water, sugarcane juice, energy drink, sports drinks	1.3
Canned vegetables	Olives, canned peas, canned corn, sauerkraut, canned hearts of palm, mushrooms	1.1
Not included in a food category	Coconut milk, guarana powder, barley powder, diet shake powder, protein supplement	0.7

Table S2. Geographic region distribution according to food security status based on the Household Budget Survey 2017-2018, Brazil [1].

Geographic region	Brazilian Food Insecurity Scale (EBIA)						p [2]
	Food security		Mild food insecurity		Moderate or severe food insecurity		
	%	(95% CI)	%	(95% CI)	%	(95% CI)	
North	37.9	(34.3, 41.8)	32.7	(29.2, 36.5)	29.3	(25.5, 33.3)	<0.001
Northeast	46.6	(44.7, 48.5)	31.9	(30.3, 33.6)	21.5	(19.9, 23.0)	
Southeast	65.0	(62.3, 67.6)	26.4	(24.1, 29.0)	8.6	(7.4, 9.9)	
South	76.9	(74.1, 79.4)	18.1	(15.9, 20.6)	4.9	(3.9, 6.2)	
Midwest	63.3	(59.9, 66.6)	25.3	(22.5, 28.3)	11.4	(9.4, 13.8)	

[1] All the analyses took into account the sampling survey design.
[2] Differences of frequencies (%) across food security status were evaluated using Pearson's Chi-square test adjusted for the complex sampling design.
